# An all ambient, room temperature–processed solar cell from a bare silicon wafer

**DOI:** 10.1093/pnasnexus/pgad067

**Published:** 2023-03-14

**Authors:** Kazuya Okamoto, Yutaka Fujita, Kosuke Nishigaya, Katsuaki Tanabe

**Affiliations:** Department of Chemical Engineering, Kyoto University, Nishikyo, Kyoto 615-8510, Japan; Department of Chemical Engineering, Kyoto University, Nishikyo, Kyoto 615-8510, Japan; Department of Chemical Engineering, Kyoto University, Nishikyo, Kyoto 615-8510, Japan; Department of Chemical Engineering, Kyoto University, Nishikyo, Kyoto 615-8510, Japan

**Keywords:** photovoltaics, PEDOT:PSS, surface, interface, low cost

## Abstract

Solar cells are a promising optoelectronic device for the simultaneous solution of energy resource and environmental problems. However, their high cost and slow, laborious production process so far severely hinder a sufficient widespread of clean, renewable photovoltaic energy as a major alternative electricity generator. This undesirable situation is mainly attributed to the fact that photovoltaic devices have been manufactured through a series of vacuum and high-temperature processes. Here we realize a poly(3,4-ethylenedioxythiophene)–poly(styrenesulfonate) (PEDOT:PSS)/Si heterojunction solar cell fabricated only in ambient and room temperature conditions from a plain Si wafer, with an over 10% energy conversion efficiency. Our production scheme is based on our finding that PEDOT:PSS photovoltaic layers actively operate even on highly doped Si substrates, which substantially mitigates the condition requirements for electrode implementation. Our approach may pave the way for facile, low-cost, high-throughput solar cell fabrication, useful in various fields even including developing countries and educational sites.

**Significance Statement**

To the best of our knowledge, this study presents the first solar cell fabricated through all ambient air and room temperature conditions from a plain Si wafer. This solar cell has exhibited an energy conversion efficiency of over 10%. We have found that PEDOT:PSS photovoltaic layers actively operate even on highly doped Si substrates, which substantially mitigates the condition requirements for electrode implementation. This finding could be a technological key factor for the further improvement of organic–inorganic hybrid solar cells. Our approach may pave the way for facile, low-cost, high-throughput solar cell production that is effective for various applications, even including the use in rural areas or for educational purposes.

## Introduction

Solar cells, also called photovoltaics, are devices that convert sunlight energy into electricity by the photovoltaic effect discovered by the French scientist Henri Becquerel in 1839. Electron–hole pairs are generated by the energy of the incident photons overcoming the energy bandgap of the photovoltaic material to make a current flow according to the built-in potential slope in the material. Solar cells have been recognized as an important alternative power source, especially since the oil crises in the 1970s. Solar cells are also promising as a carbon-free energy source to suppress global warming. However, their high cost and slow, laborious production process so far severely hinder a sufficient wide use of clean, renewable photovoltaic energy as a major alternative electricity generator, particularly in the rural area of the world. The main reasoning factor for such an undesirable situation is the fact that the production of solar cells inevitably requires a series of controlled atmosphere or vacuum and high-temperature processes and thus accompanied expensive facilities and enormous machine process time (1–8). Therefore, simple, low-cost, and high-throughput production technologies for photovoltaic devices are highly demanded (9–12), where solution processes would be representatively preferable (9, 11, 13, 14).

Even the practically most common Si solar cell requires a doping process into a Si wafer for its photovoltaic layer formation and metal electrode deposition processes on its front and back surfaces. The doping process needs a series of heating procedures in vacuum or controlled atmosphere (e.g. inert gases) chambers. The electrode formation process accompanies multiple sintering steps in reducing atmosphere. Moreover, for the electrode formation, although electrode implementation onto electrically highly conducting Si would be easy, the base Si wafer has to be relatively insulating (i.e. doping concentration ∼10^16^ cm^−3^) for the photovoltaic layer formation (15–19). For this dilemma, the formation of a practically proper, sufficiently conducting contact at the metal electrode/Si interface requires an additional high-temperature post-annealing process after the electrode deposition. Nayak et al. processed Si cells at room temperature based on the asymmetric heterocontact concept (10), but still had to use vacuumed thermal evaporation and sputtering facilities as well as a heating pretreatment (20).

Poly(3,4-ethylenedioxythiophene)–poly(styrenesulfonate) (PEDOT:PSS)/Si heterojunction solar cells are promising for its low material cost and facile preparation (21–28). By utilizing the highly transparent and conductive PEDOT:PSS as a hole transport layer, this heterojunction provides the functionality of Schottky solar cell. Similarly to the metal–insulator–semiconductor solar cells (29, 30), this category of photovoltaics allows for simpler fabrication relative to the *p*–*n* homojunction solar cells, represented by the avoidance of the high-temperature junction diffusion step. In the present study, we find that PEDOT:PSS photovoltaic layers can be actively driven even on highly doped Si substrates (doping concentration ∼mid-10^17^ cm^−3^). Noting that this fact significantly mitigates the condition requirements for electrode implementation, we unprecedentedly fabricate a solar cell through all ambient air and room temperature conditions from a bare Si wafer, to address the issue above.

## Results and discussion

### Doping concentration of Si and interfacial conductance

**Fig. 1.**
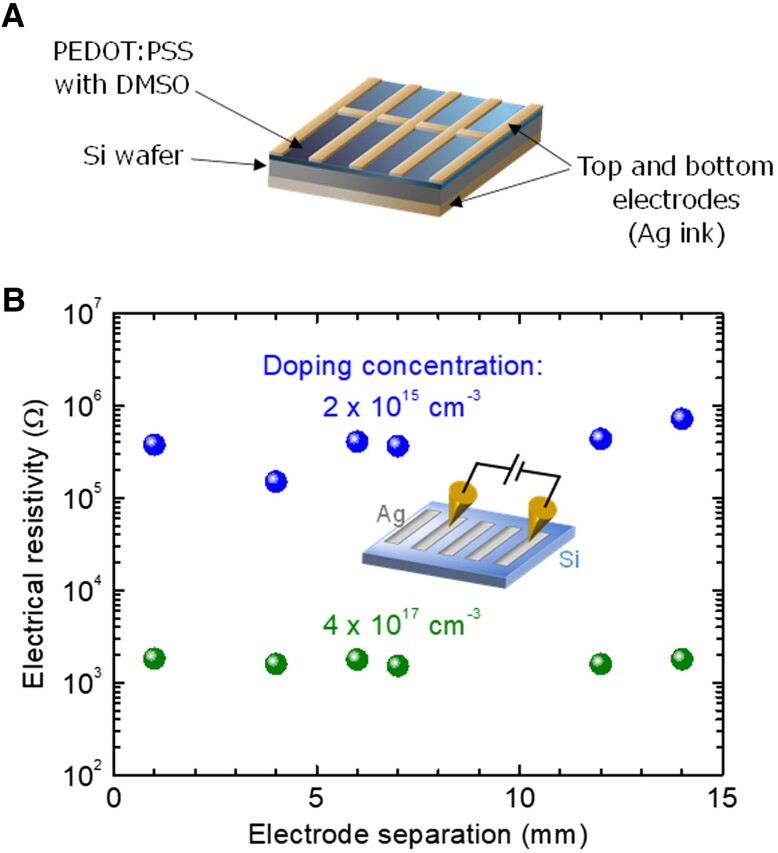
Solar cell structure and doping concentration of Si. A) Structural illustration of the PEDOT:PSS/Si heterojunction solar cell the authors fabricated in the present study. B) Electrical resistivities measured by the transmission line method for the Ag electrodes on the Si wafers with doping concentrations of 2 × 10^15^ and 4 × 10^17^ cm^−3^.

We aim to establish a low-cost and high-throughput method to fabricate solar cells under ambient temperature and pressure in this study. Figure 1A depicts a structural schematic of the PEDOT:PSS/Si heterojunction solar cell we fabricated in this study. Screen printing, electroless plating, and vacuum evaporation have been typical methods of forming electrodes for solar cells to date. However, these methods require heating and/or vacuuming processes, which inevitably cost energy, time, and thus cost. Alternatively, we employed a highly conductive Ag ink agent to simply coat the semiconductor materials as the electrodes. As a result, our solar cell fabrication process comprises only three coating processes: coatings of a PEDOT:PSS layer (i.e. *p*-type photovoltaic layer) on a Si wafer, a top Ag electrode on the PEDOT:PSS layer, and a bottom Ag electrode on the back surface of the Si wafer. We explored the optimal process condition and sequence that maximizes the solar cell performance.

It is generally difficult to obtain a low-resistivity contact suitable for device applications between a semiconductor material and a metal electrode without a post-deposition annealing process, particularly for relatively insulating semiconductors (doping concentration ∼10^16^ cm^−3^) used as the base photovoltaic layers. This fact is a crucial problem against the realization of all-room temperature fabrication of solar cells. Figure 1B presents the result of electrical measurements of the transmission line method (31–33) for the samples with Ag ink electrodes pasted onto *n*-type Si wafers of varied doping concentrations. The total series resistance is dominated by the interfacial resistance at the metal/semiconductor interfaces rather than the bulk Si resistance for both the Si wafers of doping concentrations, rationalized by the observed independence of the resistivity on the electrode separation. As observed, the contact resistivity of the electrode on the relatively insulating Si wafer (doping concentration: 2 × 10^15^ cm^−3^) is more than two orders of magnitude higher than that for the electrically conducting Si wafer (4 × 10^17^ cm^−3^). The contact resistivities were roughly determined to be 6 × 10^3^ and 50 Ω cm^2^ for the Si wafers with doping concentrations of 2 × 10^15^ and 4 × 10^17^ cm^−3^, respectively, from the plots of Fig. 1B by fitting the idealized linear equation, $R_{\mathrm{total}}=\{R_{\mathrm{bulk}}/(WD)\}L\;+\;2R_{c}/A$, where *R*_total_ is the measured total resistivity, in the unit of Ω, *R*_bulk_ is the bulk resistivity of the semiconductor in Ω cm, *W* and *D* are the effective width and depth of the current path, respectively, *L* is the electrode separation, *R*_c_ is the contact resistivity of the electrode in Ω cm^2^, and *A* is the area of the electrode. Therefore, it would be effective to unconventionally employ conducting Si wafers, to realize all room temperature and ambient manufacturing of solar cells. We are to demonstrate that such an electrically highly conductive Si wafer with a doping concentration of mid-10^17^ cm^−3^ is still able to exhibit favorable photovoltaic performance by adequately choosing the process sequence and conditions in the following.

### Fabrication process sequence

**Fig. 2.**
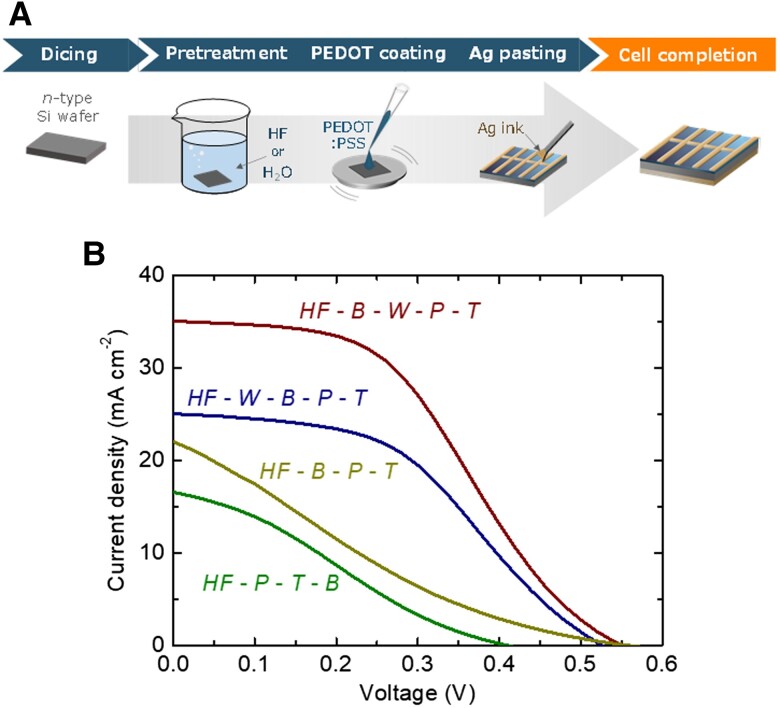
Consideration of the cell fabrication process sequence. A) Schematic flow diagram of our general fabrication scheme of the PEDOT:PSS/Si heterojunction solar cells. B) Light current–voltage characteristics of the PEDOT:PSS/Si heterojunction solar cells fabricated through the process sequences *HF* → *P* → *T* → *B*, *HF* → *B* → *P* → *T*, *HF* → *W* → *B* → *P* → *T*, and *HF* → *B* → *W* → *P* → *T*, respectively, under air mass 1.5 global, 1-sun illumination (100 mW cm^−2^).

Figure 2A shows a schematic flow diagram of our general fabrication scheme of the PEDOT:PSS/Si heterojunction solar cells. Except for the surface treatment processes, the solar cell fabrication flow can be categorized into three main processes: the coating of the PEDOT:PSS layer on the Si wafer (hereafter denoted as the process *P*), the coating of the top Ag electrode on the PEDOT:PSS layer (the process *T*), and the coating of the bottom Ag electrode on the back surface of the Si wafer (*B*). Because of the sequential restriction that the process *T* has to be carried out right after *P*, there are two potential process sequences: *P* → *T* → *B* and *B* → *P* → *T*. The green and khaki curves in Fig. 2B are the light current–voltage characteristics of the PEDOT:PSS/Si heterojunction solar cells fabricated through the process sequences *P* → *T* → *B* and *B* → *P* → *T*, respectively, after a wet hydrofluoric acid (HF) treatment (10 wt% aq, 1 min) to remove the native oxide layer on the Si surface, under air mass 1.5 global, 1-sun illumination (100 mW cm^−2^). The cell performance of the sequence *B* → *P* → *T* is observed to be somewhat higher than that of *P* → *T* → *B*. This result could be attributed to the fact that the effect of the HF pretreatment for the Si back surface is sustained better before the application of the bottom electrode. However, the severe absence of the oxide layer that acts as a potential barrier at the PEDOT:PSS/Si interface, as analyzed in the next parts, owing to the HF treatment immediately before the application of the PEDOT:PSS layer may be more plausible reasoning. Reflecting the result above, we hereafter employed the process sequence *B* → *P* → *T*.

It is known to be effective to implement a certain degree of potential barrier, such as an oxide layer, in the *p*–*n* junction (i.e. the PEDOT:PSS/Si interface) to obtain higher photovoltaic performance for PEDOT:PSS/Si heterojunction solar cells (21). We presumed that this factor becomes particularly important for the case of our cell, which employs a highly doped Si base. Therefore, next, for the process sequence *B* → *P* → *T*, we investigated the effect of an additional submergence process in deionized water to oxidize the Si surface. First, we tested a process that inserts a submergence process of the Si piece in deionized water (1 min, the process *W*) immediately after the HF treatment, *HF* → *W* → *B* → *P* → *T*. Second, we tested a process with an insertion of the water submergence between the processes *B* and *P*, resulting in the sequence *HF* → *B* → *W* → *P* → *T*. The blue and brown curves in Fig. 2B are the light current–voltage characteristics of the cells fabricated through the process sequences *HF* → *W* → *B* → *P* → *T* and *HF* → *B* → *W* → *P* → *T*, respectively. On the PEDOT:PSS/Si interface, we note in Fig. 2B that the cell performance for *HF* → *B* → *W* → *P* → *T* is higher than that for *HF* → *B* → *P* → *T*, which certifies that it is better to oxidize the top Si surface to a certain extent, as mentioned above. On the bottom Si/electrode interface, the performance for *HF* → *B* → *W* → *P* → *T* is found to be higher also than that for *HF* → *W* → *B* → *P* → *T*, which indicates that it is better not to oxidize the bottom Si surface at the same time of the oxidation of the top Si surface, to secure a high electrical conductivity at the bottom electrode contact. Overall, the process sequence *HF* → *B* → *W* → *P* → *T* is considered excellent to ideally oxidize the PEDOT:PSS/Si interface while sustaining the bottom Si/electrode interface oxide free. Therefore, we employ this optimal process sequence in the following sections.

### PEDOT:PSS emitter formation

**Fig. 3.**
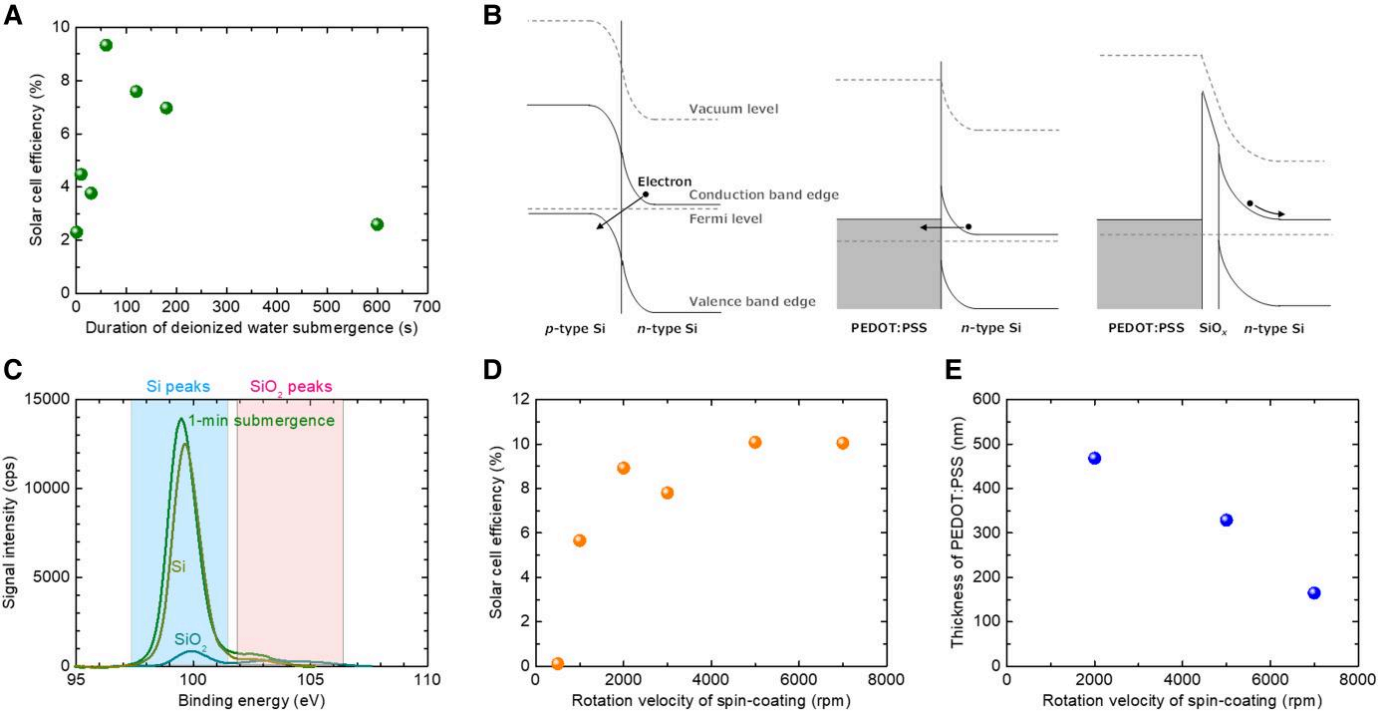
PEDOT:PSS emitter formation conditions. A) Dependence of the energy conversion efficiency of the PEDOT:PSS/Si heterojunction solar cells on the duration of the submergence process of the Si substrate in deionized water. B) Conceptual schematics of the band alignment in relation to charge separation in (left) a homojunction Si solar cell and PEDOT:PSS/Si heterojunction solar cells with highly doped Si (middle) without and (right) with a SiO*_x_* layer. C) X-ray photoelectron spectra of the Si wafer immediately after the 1-min submergence in deionized water, a Si wafer with a 2-μm-thick thermal oxide atop as a reference of SiO_2_, and an HF-treated pristine Si wafer as a reference of Si. D) Dependence of the energy conversion efficiency of the PEDOT:PSS/Si heterojunction solar cells on the rotation velocity of spin coating. E) Dependence of the thickness of the PEDOT:PSS layer on the rotation velocity of spin coating.

As observed above, the oxidation of the PEDOT:PSS/Si interface is an important factor for the solar cell performance. Therefore, we would like to determine the optimal degree of oxidation. For the process sequence *HF* → *B* → *W* → *P* → *T*, we investigate the influence of the duration of *W*, the submergence process in deionized water. Figure 3A presents the dependence of the energy conversion efficiency of the PEDOT:PSS/Si heterojunction solar cells on the duration of *W*. A drastic change in the cell efficiency on the submerging time is observed, where too shallow or intense oxidation of the top Si surface severely degrades the cell. The formation of SiO*_x_*–Si bonds is considered to pose a net positive surface dipole and leads to a favorable band alignment for charge separation (21). Conceptual schematics of the band alignment in relation to charge separation for PEDOT:PSS/Si heterojunction solar cells as well as *p*–*n* homojunction Si cells with highly doped Si are depicted in Fig. 3B. The PEDOT:PSS/Si cell with a proper SiO*_x_* layer can favorably separate carriers toward the designated electrodes owing to the formation of an inversion layer, while the homojunction Si cell and the nonoxidized PEDOT:PSS/Si cell suffer from a substantial amount of tunneling leakage current. Thicker oxide nevertheless degrades the cell performance by inevitably inducing higher series resistance. Consequently, the duration of about 1 min for the submergence in deionized water was found to be most suitable. The composition of the generated SiO*_x_* was roughly determined as *x* = 0.4 by X-ray photoelectron spectroscopy (Fig. 3C) of the Si wafer immediately after the 1-min submergence in deionized water, via a linear interpolation of the SiO_2_/Si peak area ratio between those for a Si wafer with a 2-μm-thick thermal oxide atop and an HF-treated pristine Si wafer. Note that the possibility of underestimation of *x* by the additional correction of signals from the underneath Si wafer through the very thin oxide layer in the X-ray photoelectron spectroscopy cannot be eliminated. Spectroscopic ellipsometry (300–800 nm) also determined the composition and thickness, *t*, of the SiO*_x_* layer as *x* = 0.5 and 0.9 and *t* = 1.1 and 3.5 nm for the submergence duration of 1 and 10 min in deionized water, respectively. Deducing from this data, in combination with Fig. 3A, the point of turnover in the oxide thickness on the cell efficiency is estimated to be about 1 nm. The performance of PEDOT:PSS/Si heterojunction solar cells is thus observed to be highly sensitive to the tiny thickness of the interfacial oxide. A surface passivation effect of the Si edges might also have been induced by water but is thought to be small accounting for the fact that the submergence was carried out at room temperature.

Next, we investigate the influence of the thickness of the PEDOT:PSS emitter in our heterojunction solar cell. This structural parameter is related to the process parameter of the rotation velocity in spin coating of the PEDOT:PSS solution. Figure 3D presents the dependence of the energy conversion efficiency of the PEDOT:PSS/Si heterojunction solar cells on the rotation velocity of spin coating. The cell efficiency becomes higher for higher spin velocity and is maximized around 5,000 rpm. Figure 3E plots the relationship between the thickness of the PEDOT:PSS layer, determined via cross-sectional scanning electron microscopy, and the rotation velocity of spin coating. The optimal spin velocity of 5,000 rpm thus provides a PEDOT:PSS thickness of ∼300 nm. The trend found in Figs. 3D and E thus indicates that the PEDOT:PSS emitter layer had better be relatively thin in order to acquire high energy conversion efficiency. A thicker PEDOT:PSS emitter would increase the optical loss by its own absorption, and a thinner one would increase the electrical resistivity in the lateral direction toward the top electrode and/or miss a sufficient thickness of the inversion layer. Incidentally, although we processed (8 mm)^2^ square Si pieces in this preliminary laboratory-scale test, our fabrication scheme is straightforwardly scalable for industrialization, as highly uniform spin coating of organic materials onto 6-inch Si wafers has been well established, with typical thickness deviations of <5% (34, 35).

### Solar cell characterization and perspective

**Fig. 4.**
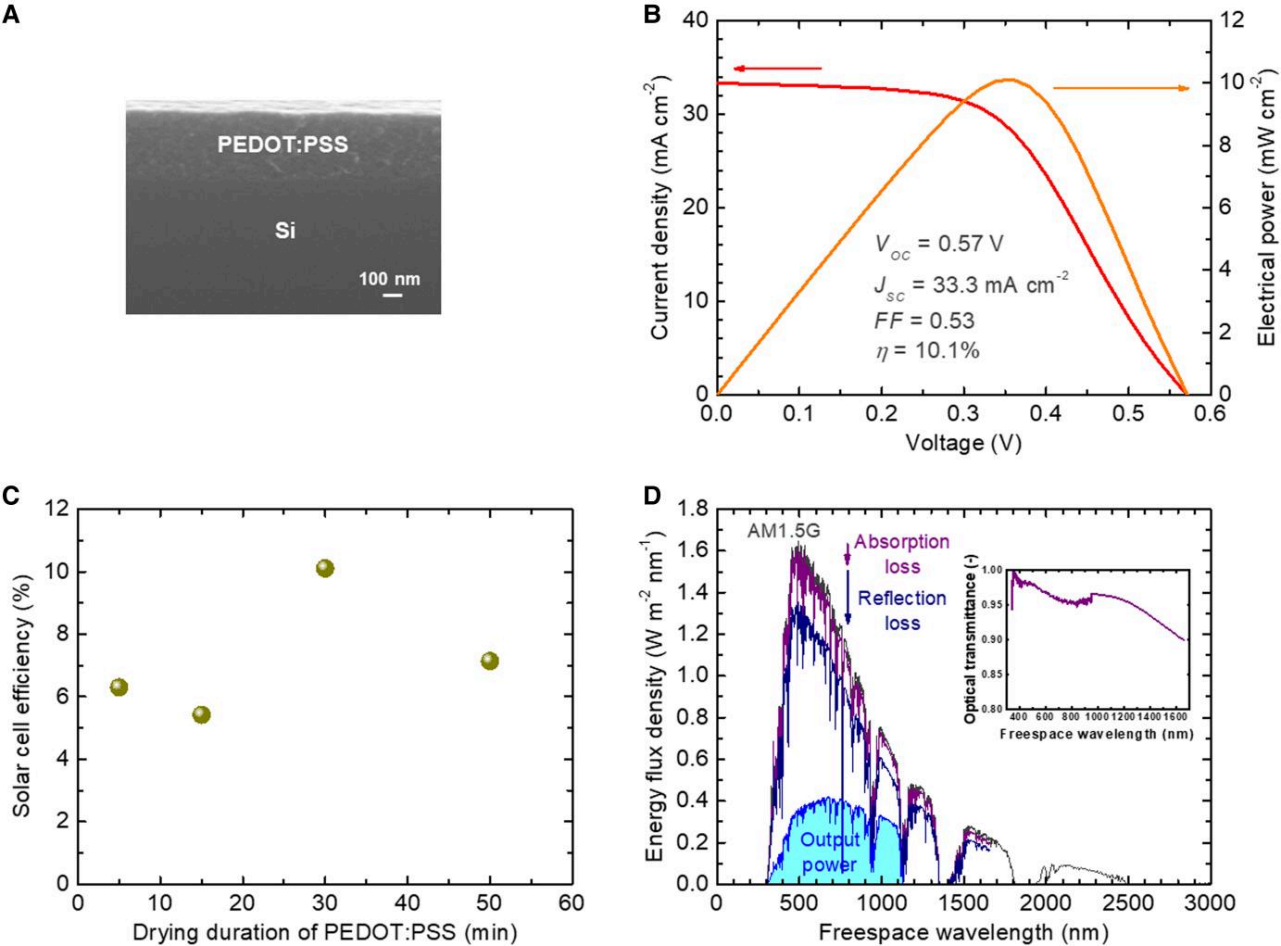
PEDOT:PSS/Si heterojunction solar cell characterization. A) Cross-sectional scanning electron microscope image of the PEDOT:PSS/Si heterojunction solar cell, focusing on the PEDOT:PSS/Si interface. C) Light current–voltage and power–voltage characteristics of the PEDOT:PSS/Si heterojunction solar cell under air mass 1.5 global, 1-sun illumination (100 mW cm^−2^). *V*_OC_, *J*_SC_, *FF*, and *η* denote the open-circuit voltage, short-circuit current density, fill factor, and energy conversion efficiency, respectively. C) Dependence of the energy conversion efficiency of the PEDOT:PSS/Si heterojunction solar cell on the drying duration of the PEDOT:PSS layer at room temperature in ambient air between its spin coating and the electrode application. D) Air mass 1.5 global, 1-sun solar spectrum, that with the absorption loss in the PEDOT:PSS layer, that further with the reflection loss at the PEDOT:PSS/Si interface, corresponding to the optical incidence into Si, and the detailed balance limit output of Si solar cells. AM1.5G stands for the air mass 1.5 global solar spectrum. Inset shows the measured transmission spectrum of the PEDOT:PSS layer.

Finally, we present the result from the optimal fabrication condition with the process sequence of *HF* → *B* → *W* → *P* → *T*, an oxidizing submergence process in deionized water for 1 min and a rotation velocity of 5,000 rpm in the spin coating of PEDOT:PSS. Figure 4A presents a cross-sectional scanning electron microscope image of the fabricated PEDOT:PSS/Si heterojunction solar cell, focusing on the PEDOT:PSS/Si interface. Figure 4B presents the light current–voltage and power–voltage characteristics of the cell under air mass 1.5 global, 1-sun illumination (100 mW cm^−2^) by a solar simulator. An energy conversion efficiency over 10% is thus obtained, with an open-circuit voltage of 0.57 V, a short-circuit current density of 33.3 mA cm^−2^, and a fill factor of 0.53, resulting in an efficiency of 10.1%. As the state-of-the-art performance of PEDOT:PSS/Si heterojunction solar cells, energy conversion efficiencies of 16–17% have been achieved by using vacuum and high-temperature processes (27, 28, 36, 37). Our efficiency positions at significantly lower relative to those values, due to the severe restriction of the process condition. The low fill factor severely limiting the efficiency is attributed to the relatively high series resistivity. It is known that the residual water content in the PEDOT:PSS layer interacting with defects and air gaps at the PEDOT:PSS/Si interface could form dipoles to cause a significant resistance (38, 39). Nevertheless, as the dependence of our efficiency on the drying duration shows in Fig. 4C, long exposure of the PEDOT:PSS material in ambient air may rather result in irreversible structural degradation of the polymer chains by fast oxidation promoted by oxygen and moisture (39, 40). Therefore, to circumvent such a trade-off, an annealing treatment at above 100°C in vacuum or nonoxidizing atmosphere might be required for the sufficient removal of moisture and improvement of crystallinity, to improve the solar cell performance further (39, 40). As we analyzed the dark current–voltage characteristics of the cell, the diode ideality factor was slightly lower than 2 in the vicinity of zero bias voltage and 3–4 above 0.1 V. This result indicates that the diode characteristic of the cell is limited by the series resistance, beyond the recombination current regime. In the total series resistance of the cell, the relative contribution of the top contact, PEDOT:PSS bulk (mainly for the horizontal transport), PEDOT:PSS/Si interface, Si bulk, and bottom contact were determined approximately as 10:20:30:0:40%, respectively, through independent transmission line method measurements. In addition, the built-in potential of the junction was estimated to be 0.60 V by dark current–voltage analysis via the fitted saturation current density assuming a Schottky-type interface. Relative to the open-circuit voltage of 0.57 V, this result indicates a voltage loss of 0.03 V at the bottom Si/electrode interface. Therefore, reduction of the bottom contact resistance is of primary importance for the device performance improvement, for instance by further optimization of the surface pretreatment or doping concentration of Si. While higher doping concentrations provide higher contact conductivities as demonstrated in this study, too heavy doping would rather decrease the cell performance due to the shorter minority carrier life time and insufficient depletion layer thickness (41, 42). Let us roughly estimate the ideal performance of the cell. Figure 4D plots the air mass 1.5 global, 1-sun solar spectrum, accompanied with those subtracted by a partial absorption in the PEDOT:PSS layer and further by a partial reflection at the PEDOT:PSS/Si interface, corresponding to the optical incidence into Si. The absorption loss was simulated via the transmission spectrum of the PEDOT:PSS layer prepared on a glass plate in the same manner as that of the solar cell, measured by subtracting that of the plain glass plate without PEDOT:PSS, as presented in the inset of Fig. 4D. The reflection loss was estimated as 16% from the refractive indices of PEDOT:PSS (43, 44) and Si of 1.5 and 3.5, respectively. Also plotted in Fig. 4D is the simulated fraction of the solar spectrum a Si cell can ideally output, by the detailed balance limit calculation method (45), resulting in an efficiency of 24.2%. The ideal short-circuit current density was calculated to be 35.8 mA cm^−2^. Comparing this value with our data of 33.3 mA cm^−2^, the average internal quantum efficiency in Si of our cell is roughly determined to be 93%. It is thus thought that our cell already exerts nearly its full potential for the current output. Nevertheless, application of antireflective coating and firm surface passivation would still increase the cell performance by a certain extent. The efficiencies of the fabricated cells were observed to decrease by about half in 1 h in ambient air presumably due to partial oxidation of PEDOT:PSS (46, 47), and therefore, proper packaging may be necessary in practical use, except for the educational purpose. In addition, emerging alternative hole transport materials with higher stability, such as PEDOT:F (12), could be utilized. This device is the first solar cell fabricated only in ambient and room temperature conditions from a bare Si wafer, to the best of our knowledge. Our fabrication approach may lead to convenient, low-cost, high-throughput photovoltaic device manufacture, particularly beneficial for the use in developing countries and educational sites. In addition, this finding itself could be a technological key factor for the further improvement of organic–inorganic hybrid solar cells (48–53).

## Conclusions

In this study, we unprecedentedly fabricated a solar cell, from a bare Si wafer, through all ambient air and room temperature conditions. Our device structure was based on a PEDOT:PSS/Si heterojunction that we found active even for a highly doped Si substrate, which significantly eases the electrode implementation. This PEDOT:PSS/Si heterojunction solar cell exhibited an energy conversion efficiency of over 10%. The presented engineering scheme could pave the way for easy, low-cost, high-throughput solar cell production that is effective for various applications, even including the use in rural areas or for educational purposes.

## Methods

### Solar cell fabrication

The whole experimental processes of this study were conducted in ambient air without a chamber (e.g. a vacuum chamber or a grove box) or heating, in a noncleanroom, regular experimental room with a particle density of ∼5 million m^−3^, as measured by a regular particle counter. Single-side-polished *n*-type Si wafers (thickness: 280 μm; crystalline plane orientation: <100>; dopant: phosphorus; doping concentration: 4 × 10^17^ cm^−3^) were used. The Si wafer was diced into (8 mm)^2^ square pieces. The Si piece was then subject to a wet HF treatment (10 wt% aq, 1 min) to remove the native oxide layer on the Si surface. Dimethyl sulfoxide (Fujifilm Wako Pure Chemical Corp.) was mixed into PEDOT:PSS aqueous solution (3.0–4.0 w/v%; Sigma-Aldrich Corp.) to a concentration of 5 v/v%, to enhance the electrical conductivity of PEDOT:PSS (54). This PEDOT:PSS solution was subsequently spin coated onto the polished side surface of the Si piece with a designated rotation velocity [basically 2,000 rpm (26), unless otherwise noted] for 1 min. The sample piece was left for 30 min, unless otherwise noted, at room temperature in ambient air to dry the PEDOT:PSS layer. Ag ink (*DOTITE* D-362; Fujikura Kasei Corp.) was then pasted on the top and bottom surfaces of the PEDOT:PSS-coated Si piece as electrodes of a solar-cell device. The thickness of the applied electrodes was 15–20 μm, as observed in cross-sectional scanning electron microscopy. No antireflective coating was applied on the top surface. For some experiments, the sequence of the implementation of the PEDOT:PSS layer and the electrodes was varied, and a submergence process of the Si piece in deionized water was partly inserted.

### Characterization

The doping concentrations, bulk resistivities, and mobilities of the Si wafers were determined by Hall effect measurement systems (HL5500PC, Accent Corp. and ResiTest 8300, Toyo Corp.) in two individual institutes. For the electrical measurement of the transmission line method, the diced Si wafers with doping concentrations of 2 × 10^15^ and 4 × 10^17^ cm^−3^ (bulk resistivity: 2 and 3 × 10^–2^ Ω cm and mobility: 1 × 10^3^ and 5 × 10^2^ cm^2^ V^–1^ s^−1^, respectively) were subject to the wet HF treatment (10 wt% aq, 1 min). The Ag ink was then pasted onto the back surface of the Si piece through a shadow mask with an array of slits to form a stripe of electrodes with an area of 1 × 6 mm with designated separation distances (31–33). The measurement configuration is depicted in the inset of Fig. 1B. The current–voltage characteristics of the circuit through Si between the two electrodes were measured by using an electrical source measure unit (Model 2400; Keithley Instruments Inc.).

The microscopic observation of the solar cell samples was carried out by using a field emission scanning electron microscope (JSM-6700F; JEOL Ltd.). The light current–voltage characteristics of the solar cells were measured by using a solar simulator (XES-40S1; San-Ei Electric Corp.) and the Keithley 2400 source measure unit in the four-probe configuration. The illumination source of the solar simulator was a xenon (Xe) lamp filtered to achieve a simulated air mass 1.5 global spectrum. The areally uniform irradiation intensity at the plane of the solar cell sample under test was adjusted to 100 mW cm^−2^ (the 1-sun condition) by using a standard Si cell (Reference PV Cell AK-200; Konica Minolta Inc.) calibrated and certified by the National Institute of Advanced Industrial Science and Technology. During the light current–voltage measurement, the temperature of the solar cell sample was held at 25°C by using a sample stage equipped with a Peltier temperature controller.

## Data Availability

All data are included in the manuscript and/or supporting information.
